# Non-equilibrium Model for Nanofluid Free Convection Inside a Porous Cavity Considering Lorentz Forces

**DOI:** 10.1038/s41598-018-33079-6

**Published:** 2018-11-15

**Authors:** M. Sheikholeslami, Ilyas Khan, I. Tlili

**Affiliations:** 1 Faculty of Mathematics and Statistics, Ton Duc Thang University, Ho Chi Minh City, Vietnam; 2 Department of Mechanical Engineering, Babol Noshirvani University of Technology, Babol, Iran; 3 Energy and Thermal Systems Laboratory, National Engineering School of Monastir, Street Ibn El Jazzar, 5019 Monastir, Tunisia

## Abstract

In current article, transportation of CuO nanoparticles through a porous enclosure is demonstrated. The enclosure has complex shaped hot wall. Porous media has been simulated via two temperature equations. Magnetic force impact on nanofluid treatment was considered. Control volume based finite element method has been described to solve current article in vorticity stream function form. Single phase model was chosen for nanofluid. Nanofluid characteristics are predicted via KKL model. Roles of solid-nanofluid interface heat transfer parameter (Nhs), porosity, Hartmann and Rayleigh numbers have been illustrated. Outputs illustrated that conduction mode reduces with augment of Ra. Increasing magnetic forces make nanofluid motion to decrease. Temperature gradient of nanofluid decreases with augment of Nhs. Reducing porosity leads to enhance in Nusselt number.

## Introduction

Solar power collectors and drying technologies are two common uses for porous enclosure. In some application, researchers should consider two- temperature model. Alsabery *et al*.^1^ demonstrated nanoparticletransportation in a tilted porous cavity. They indicated that convective flow is significantly influenced by the permeable layer augmentation. Lu *et al*.^2^ investigated about nanofluid radiative three dimensional flow containing gyrotactic microorganism with anisotropic slip. They considered the effect of activation energy. Zaimi *et al*.^3^ illustrated nanofluid boundary layer movement on a porous plate. Sheikholeslami and Shehzad^4^ showed the two temperature model for nanoparticle migration inside a permeable medium. They revealed that Nu increases with decrease of porosity. Khan *et al*.^5^ reported the nanofluid mixed convection over an oscillating vertical plate. They utilized Laplace transform method to solve the governing equations.

Sheikholeslami and Shehzad^6^ investigated the role of radiation on nanoparticle treatment. They found that Nu decreases with reduce of radiation parameter. Haq *et al*.^7^ used carbon nanotubes to improve convective heat transfer over plate with slip flow. Carbon Nanotubes has been dispersed in to engine oil by Haq *et al*.^8^ is examined of magnetic forces. Tripathi *et al*.^9^ illustrated viscous dissipation and Hall effects on nanofluid rotating flow. Selimefendigil and Oztop^10^ depicted impact of inclination on hydrothermal behavior. They found that titled angle can be used as control parameter.

Promvonge *et al*.^11^ applied new way in a duct to improve the thermal characteristics. Sheikholeslami^12^ described the impact of electric filed on nanofluid free convection. He proved that Nusselt number enhances by adding electric field. Aman *et al*.^13^ illustrated the nanofluid thermal improvement in migration of CNTs nanoparticles. Sheikholeslami and Seyednezhad^14^ illustrated nanofluid Electrohydrodynamic flow in a permeable enclosure. Different applications of Fe3O4-water nanofluid were categorized by Sheikholeslami and Rokni^15^. Akbar *et al*.^16^ showed the role of Hartmann flow on nanoparticles migration in a duct. Najib *et al*.^17^ demonstrated the impact of chemical factor on flow style. They found that the Nu augments with augment of curvature. Various articles were been available about nanoparticle migration through porous media^18–21^.

Current publication is about nanoparticle migration in a porous enclosure with two temperature model via CVFEM considering magnetic force. Results illustrate the roles of significant parameters on contours.

## Explanation of Geometry

**Figure 1:**
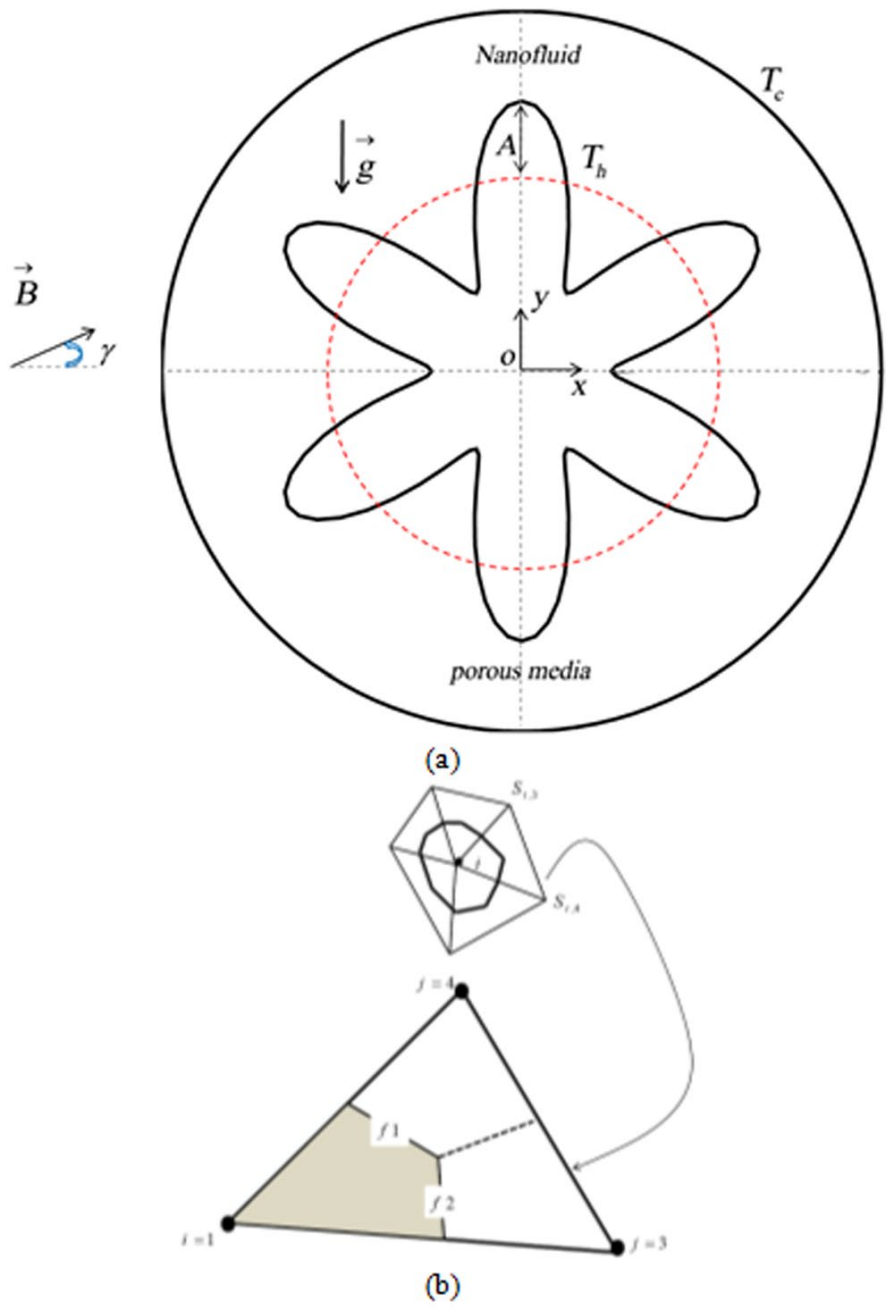
(**a**) Geometry and the boundary conditions with (**b**). A sample triangular element and its corresponding control volume.

Figure 1 illustrates the details of current geometry. The hot wall can formulate by:

1 $${r}_{out}={r}_{in}+A\,\cos \,(N\,(\zeta -{\zeta }_{0}))$$

*r*_*out*_, *r*_*in*_, *A*, *N* are outer and inner radius, amplitude, number of undulation. The porous enclosure is full of nanofluid and influenced by magnetic force.

## CVFEM and Explanation

### Formulation

According to existence of magnetic force and two temperature model for porous medium the basic formulas are:

2 $$\frac{\partial u}{\partial x}+\frac{\partial v}{\partial y}=0$$

3 $$-\frac{\partial P}{\partial x}+{\sigma }_{nf}{B}_{0}^{2}[v\,(\sin \,\gamma )(\cos \,\gamma )-u{(\sin \gamma )}^{2}]-\frac{{\mu }_{nf}}{K}u=0$$

4 $$\begin{array}{rcl}\frac{{\mu }_{nf}}{K}v & = & -\frac{\partial P}{\partial y}+(T-{T}_{c})g{\rho }_{nf}{\beta }_{nf}\\  &  & +{\sigma }_{nf}{B}_{0}^{2}[u(\cos \,\gamma \,)(\sin \,\gamma )-v{(\cos \gamma )}^{2}]\end{array}$$

5 $$\frac{1}{\varepsilon }(u\frac{\partial {T}_{nf}}{\partial x}+v\frac{\partial {T}_{nf}}{\partial y})=\frac{{h}_{nfs}}{(\varepsilon ){(\rho {C}_{p})}_{nf}}(-{T}_{nf}+{T}_{s})+\frac{{k}_{nf}}{{(\rho {C}_{p})}_{nf}}(\frac{{\partial }^{2}{T}_{nf}}{\partial {y}^{2}}+\frac{{\partial }^{2}{T}_{nf}}{\partial {x}^{2}})$$

6 $$\frac{{k}_{s}}{{(\rho {C}_{p})}_{s}}(\frac{{\partial }^{2}{T}_{s}}{\partial {x}^{2}}+\frac{{\partial }^{2}{T}_{s}}{\partial {y}^{2}})+\frac{{h}_{nfs}}{(1-\varepsilon ){(\rho {C}_{p})}_{s}}({T}_{nf}-{T}_{s})=0$$

(*ρC*_*p*_)_*nf*_, (*ρβ*)_*nf*_, *ρ*_*nf*_, *σ*_*nf*_ and *k*_*nf*_, *μ*_*nf*_ can define as^22^:

7 $${(\rho {C}_{p})}_{nf}=\varphi {(\rho {C}_{p})}_{p}+(1-\varphi ){(\rho {C}_{p})}_{f}$$

8 $${(\rho \beta )}_{nf}=(1-\varphi ){(\rho \beta )}_{f}+\varphi {(\rho \beta )}_{p}$$

9 $${\rho }_{nf}={\rho }_{f}(1-\varphi )+{\rho }_{p}\varphi $$

10 $$\frac{{\sigma }_{nf}}{{\sigma }_{f}}=\frac{(MM-1)3\varphi }{(MM+2)+\varphi (1-MM)}+1,\,MM=\frac{{\sigma }_{p}}{{\sigma }_{f}}$$

11 $$\begin{array}{rcl}\frac{{k}_{nf}}{{k}_{f}} & = & 1-3\frac{(1-\frac{{k}_{p}}{{k}_{f}})\varphi }{(1-\frac{{k}_{p}}{{k}_{f}})\varphi +(\frac{{k}_{p}}{{k}_{f}}+2)}+\sqrt{\frac{{\kappa }_{b}T}{{\rho }_{p}{d}_{p}}}{c}_{p,f}(5\times {10}^{4})g^{\prime} ({d}_{p},\,T,\,\varphi ){\rho }_{f}\varphi \\ g^{\prime} ({d}_{p},\,T,\,\varphi ) & = & ({a}_{2}Ln({d}_{p})+{a}_{1}+{a}_{3}Ln(\varphi )+{a}_{5}Ln{({d}_{p})}^{2}+{a}_{4}Ln({d}_{p})Ln(\varphi ))Ln(T)\\  &  & +({a}_{9}Ln({d}_{p})Ln(\varphi )+{a}_{10}Ln{({d}_{p})}^{2}+{a}_{7}Ln({d}_{p})+{a}_{6}+{a}_{8}Ln(\varphi ))\end{array}$$

12 $${\mu }_{nf}=\frac{{\mu }_{f}}{{(1-\varphi )}^{2.5}}+\frac{{\mu }_{f}}{{k}_{f}}({k}_{Brownian}/\Pr )$$

where *ϕ* is nanofluid volume fraction. Required characteristics and parameters are illustrated in Tables 1 and 2^22^.

**Table 1:** The coefficient values of *CuO*−Water nanofluid.

*Coefficient values*	*CuO*−Water
*a*_1_	*−26.593310846*
*a*_2_	*−0.403818333*
*a*_3_	*−33.3516805*
*a*_4_	*−1.915825591*
*a*_5_	*6.42185846658E-02*
*a*_6_	*48.40336955*
*a*_7_	*−9.787756683*
*a*_8_	*190.245610009*
*a*_9_	*10.9285386565*
*a*_10_	*−0.72009983664*

**Table 2:** Thermo physical properties of water and nanoparticles.

	*ρ*(*kg*/*m*^3^)	*C*_*p*_(*j*/*kgk*)	*k*(*W*/*m*.*k*)	*β* × 10^5^(*K*^−1^)	*d*_*p*_(*nm*)	*σ*(Ω⋅m)^−1^
Water	*997.1*	*4179*	*0.613*	21	—	*0*.*05*
*CuO*	*6500*	*540*	*18*	*29*	*29*	*10* ^*−10*^

Considering following definitions:

13 $$\begin{array}{rcl}v & = & -\frac{\partial \psi }{\partial x},(X,\,Y)=(x,\,y)/L,\,u=\frac{\partial \psi }{\partial y},\\ {\theta }_{nf} & = & ({T}_{nf}-{T}_{c})/({T}_{h}-{T}_{c}),{\rm{\Psi }}=\psi /{\alpha }_{nf},\,{\theta }_{s}=({T}_{s}-{T}_{c})/({T}_{h}-{T}_{c}),\end{array}$$

Final equations are:

14 $$\begin{array}{rcl}\frac{{\partial }^{2}{\rm{\Psi }}}{\partial {X}^{2}}+\frac{{\partial }^{2}{\rm{\Psi }}}{\partial {Y}^{2}} & = & -\,\frac{{A}_{6}}{{A}_{5}}Ha[\frac{{\partial }^{2}{\rm{\Psi }}}{\partial {Y}^{2}}({\sin }^{2}\gamma )+\frac{{\partial }^{2}{\rm{\Psi }}}{\partial {X}^{2}}({\cos }^{2}\gamma )+2\frac{{\partial }^{2}{\rm{\Psi }}}{\partial X\,\partial Y}(\sin \,\gamma )(\cos \,\gamma )]\\  &  & -\frac{{A}_{3}\,{A}_{2}}{{A}_{4}\,{A}_{5}}\frac{\partial {\theta }_{nf}}{\partial X}Ra\end{array}$$

15 $$\varepsilon (\frac{{\partial }^{2}{\theta }_{nf}}{\partial {Y}^{2}}+\frac{{\partial }^{2}{\theta }_{nf}}{\partial {X}^{2}})+Nhs({\theta }_{s}-{\theta }_{nf})=-\,\frac{\partial {\theta }_{nf}}{\partial Y}\frac{\partial {\rm{\Psi }}}{\partial X}+\frac{\partial {\rm{\Psi }}}{\partial Y}\frac{\partial {\theta }_{nf}}{\partial X}$$

16 $$\varepsilon (\frac{{\partial }^{2}{\theta }_{s}}{\partial {Y}^{2}}+\frac{{\partial }^{2}{\theta }_{s}}{\partial {X}^{2}})+Nhs\,{\delta }_{s}({\theta }_{nf}-{\theta }_{s})=0$$

where:

17 $$\begin{array}{rcl}{A}_{1} & = & \frac{{\rho }_{nf}}{{\rho }_{f}},\,Ra={(\rho \beta )}_{f}\frac{Kg\,L\,{\rm{\Delta }}T}{{\alpha }_{f}{\mu }_{f}\,},\,{A}_{5}=\frac{{\mu }_{nf}}{{\mu }_{f}},\\ {A}_{2} & = & \frac{{(\rho {C}_{P})}_{nf}}{{(\rho {C}_{P})}_{f}},{\delta }_{s}={k}_{nf}/[{k}_{s}(1-\varepsilon )]{A}_{4}=\frac{{k}_{nf}}{{k}_{f}},\\ {A}_{3} & = & \frac{{(\rho \beta )}_{nf}}{{(\rho \beta )}_{f}},\,Ha=\frac{{\sigma }_{f}K\,{B}_{0}^{2}}{{\mu }_{f}},\\ Nhs & = & {h}_{nfs}{L}^{2}/{k}_{nf},\,{A}_{6}=\frac{{\sigma }_{nf}}{{\sigma }_{f}},\end{array}$$

Boundary conditions are:

18 $$\begin{array}{c}{\theta }_{nf}={\theta }_{s}=0.0\,\,{\rm{on}}\,{\rm{outer}}\,{\rm{wall}}\\ {\rm{\Psi }}=0.0\,\,\,\,{\rm{on}}\,{\rm{all}}\,{\rm{walls}}\\ {\theta }_{nf}={\theta }_{s}=1.0\,\,{\rm{on}}\,{\rm{inner}}\,{\rm{wall}}\end{array}$$

*Nu*_*loc*_ and *Nu*_*ave*_ are:

19 $$N{u}_{loc}=(\frac{{k}_{nf}}{{k}_{f}})\frac{\partial {\theta }_{nf}}{\partial r}$$

20 $$N{u}_{ave}=\frac{1}{2\pi }{\int }_{0}^{2\pi }N{u}_{loc}\,dr$$

### CVFEM

The innovative in which triangular element is used and upwind method is applied for advection term is CVFEM (Fig. 1(b)). Gauss-Seidel is the name of the method which is used for final step as mentioned in ref.^23^.

## Mesh Independent Test and Validation

**Figure 2:**
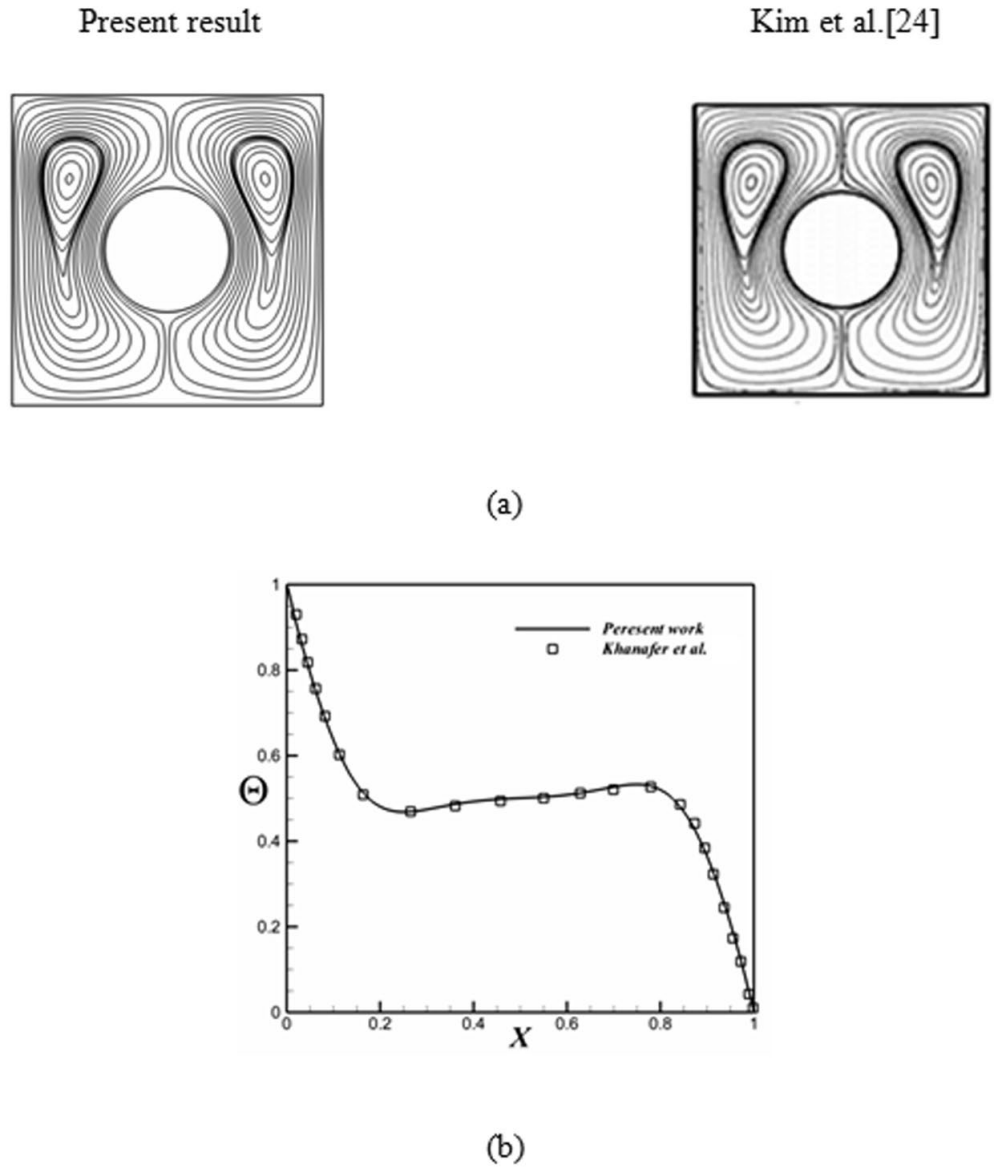
Comparison of the present solution with previous work (Kim *et al*.^24^) for different Rayleigh numbers when Ra = 10^5^, Pr = 0.7; (**b**) comparison of the temperature on axial midline between the present results and numerical results obtained by Khanafer *et al*.^25^ for *Gr* = 10^4^, *ϕ* = 0.1 and *Pr* = *6.2* (*Cu−Water*).

Obviously, the final outputs should not alter by changing mesh size. So, this test should be done for various cases as illustrated Table 3. Also, we should be sure about accuracy of written code by applying this code for previous published problem. Table 4 and Fig. 2 illustrates nice accuracy^24–26^.

**Table 3:** Comparison of the average Nusselt number *Nu*_*ave*_ for different grid resolution at *Ra* = 1000, *Ha* = 20, *ε* = 0.9, *Nhs* = 1000 and *ϕ* = 0.04.

Mesh size in radial direction × angular direction				
*61* × *181*	*71* × *211*	*81* × *241*	*91* × *271*	*101* × *301*
*1.36733*	*1.36828*	*1.36967*	*1.36971*	*1.36989*

**Table 4:** *Nu*_*ave*_for various *Gr* and *Ha* at Pr = 0.733.

*Ha*	*Gr* = 2 × 10^4^		*Gr* = 2 × 10^5^	
	Present	Rudraiah *et al*.^26^	Present	Rudraiah *et al*.^26^
0	2.5665	2.5188	5.093205	4.9198
10	2.26626	2.2234	4.9047	4.8053
50	1.09954	1.0856	2.67911	2.8442
100	1.02218	1.011	1.46048	1.4317

## Results and Discussion

In current article, migration of nanoparticle in a porous medium which is described by new porous model is presented. Numerical method is applied to display the roles of the porosity (*ε* = 0.3 *to* 0.9), Rayleigh number (*Ra* = 100,500 and 10^3^), Hartmann number (*Ha* = 0 *to* 20) and Nhs (*Nhs* = 10 *to* 1000).

**Figure 3:**
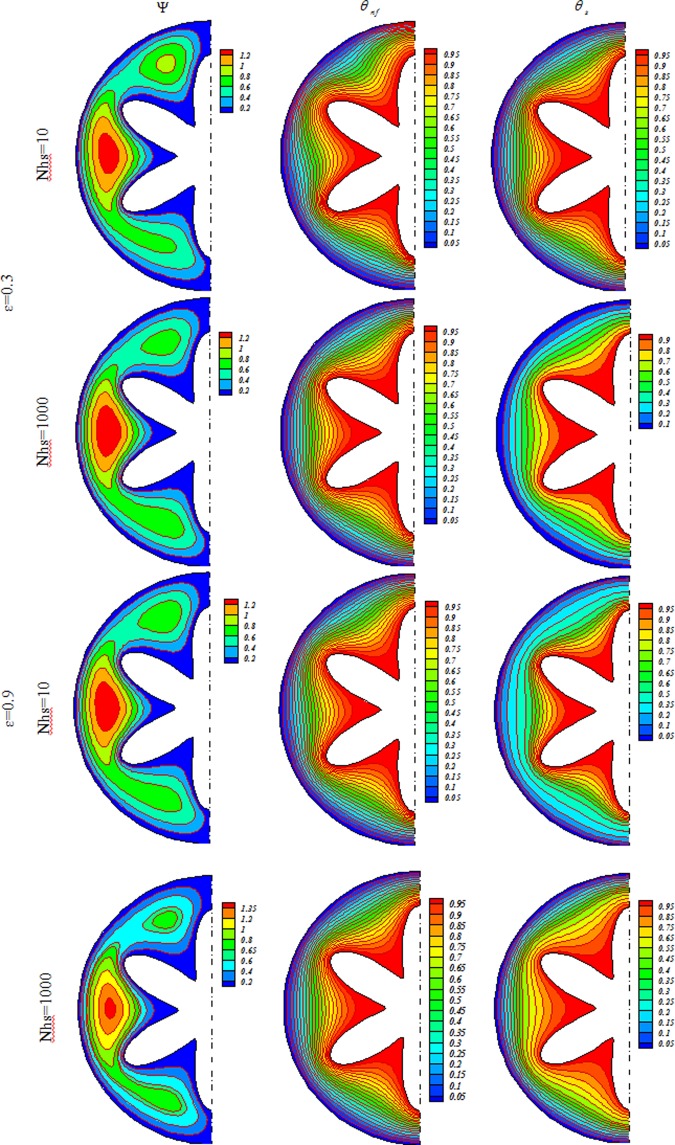
Streamlines (Ψ), isotherms for the nanofluid (*θ*_*nf*_) and the solid (*θ*_*s*_) at *Ra* = 100, *Ha* = 0, *ϕ* = 0.04.

**Figure 4:**
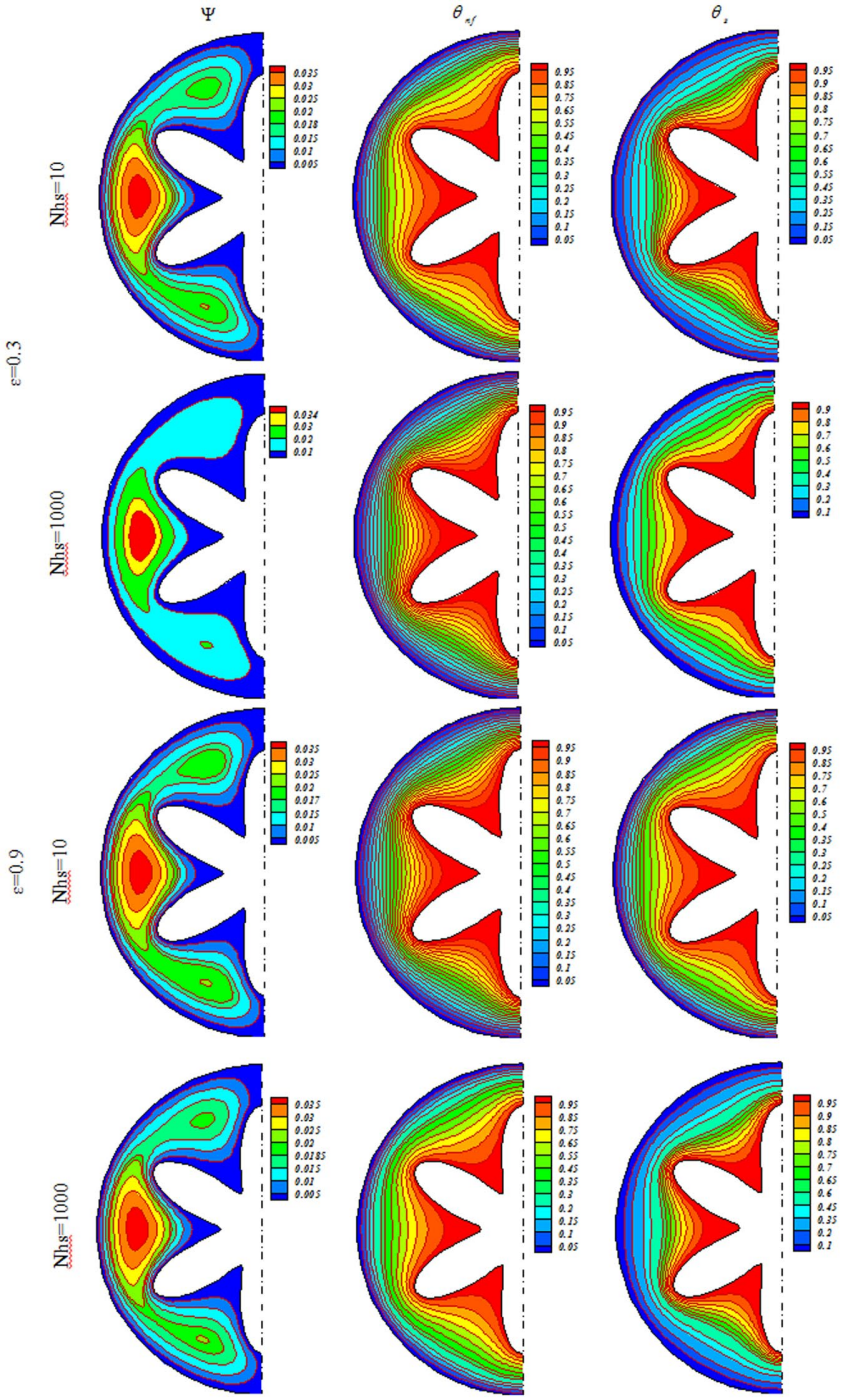
Streamlines (Ψ), isotherms for the nanofluid (*θ*_*nf*_) and the solid (*θ*_*s*_) at *Ra* = 100, *Ha* = 20, *ϕ* = 0.04.

**Figure 5:**
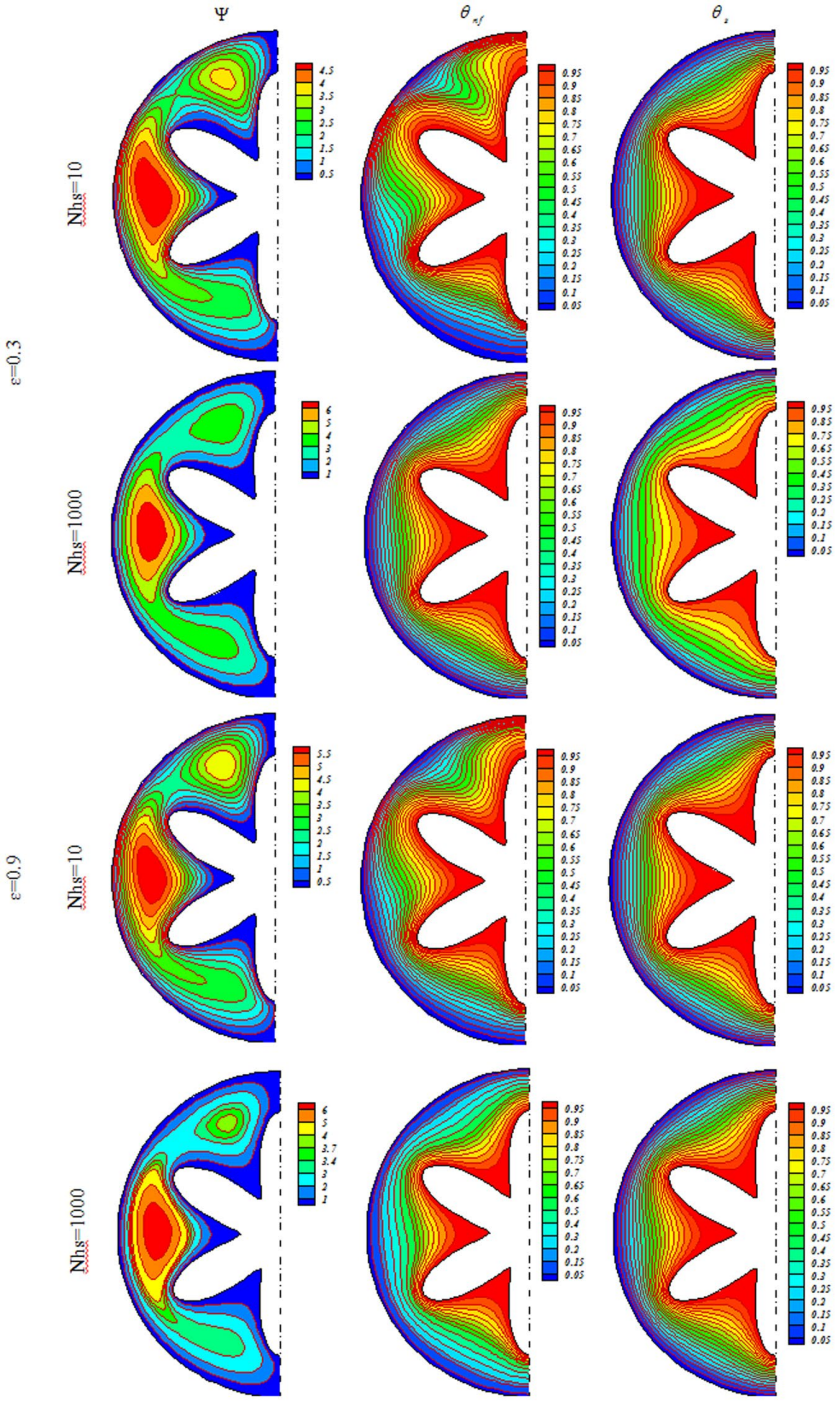
Streamlines (Ψ), isotherms for the nanofluid (*θ*_*nf*_) and the solid (*θ*_*s*_) at *Ra* = 500, *Ha* = 0, *ϕ* = 0.04.

**Figure 6:**
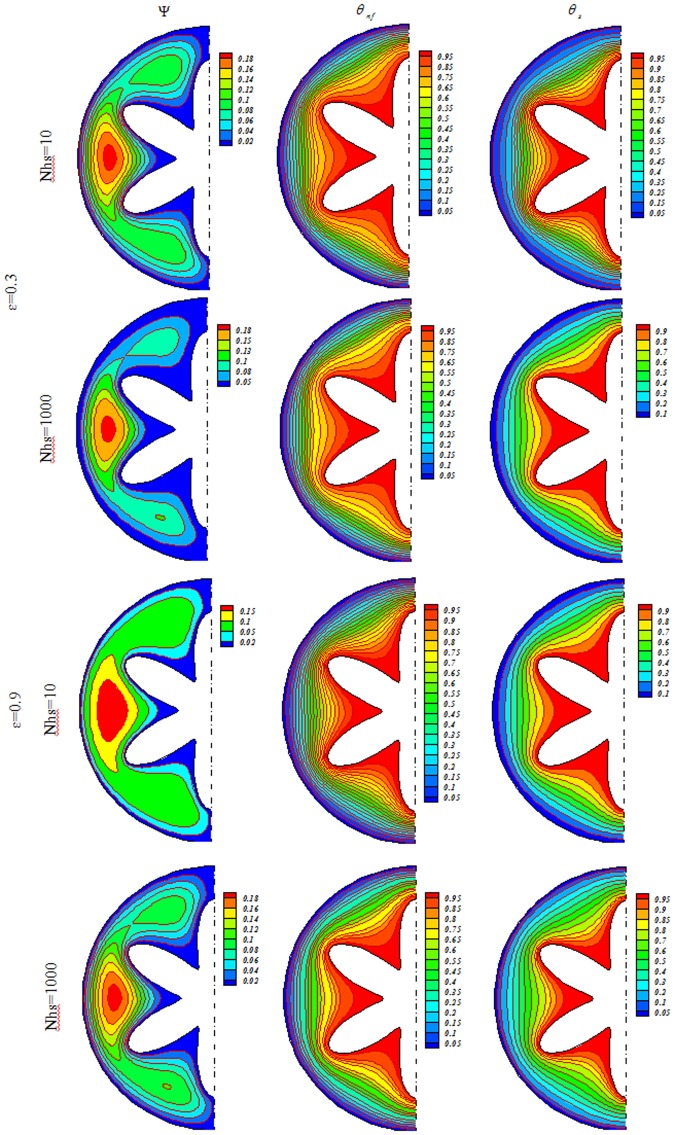
Streamlines (Ψ), isotherms for the nanofluid (*θ*_*nf*_) and the solid (*θ*_*s*_) at *Ra* = 500, *Ha* = 20, *ϕ* = 0.04.

**Figure 7:**
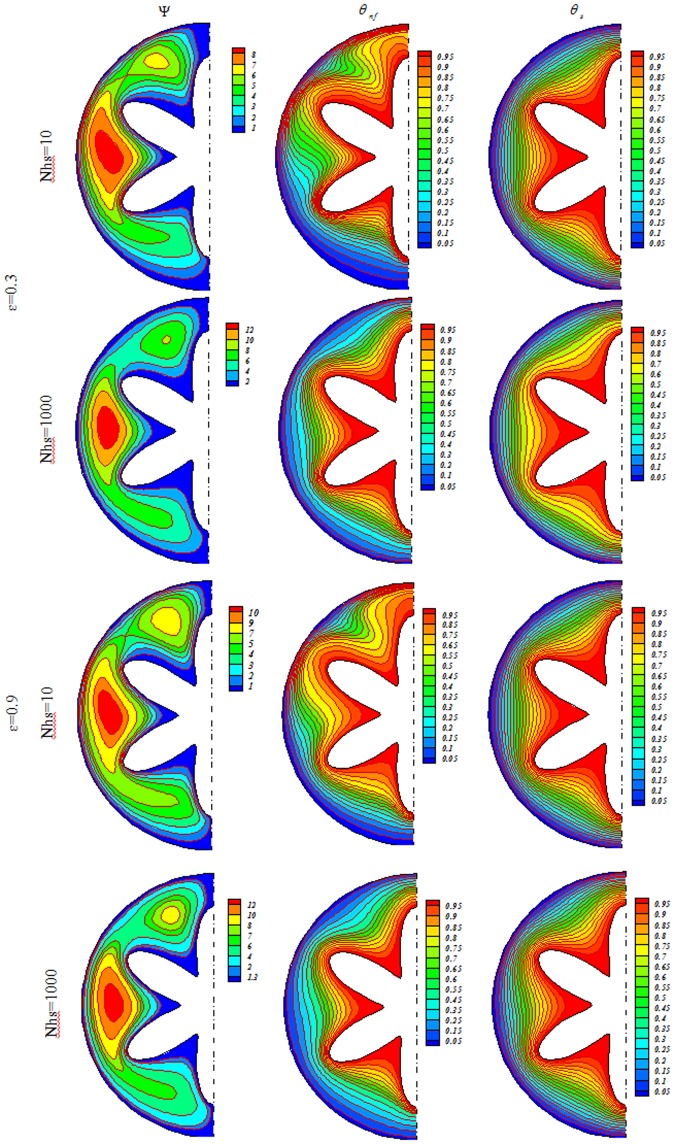
Streamlines (Ψ), isotherms for the nanofluid (*θ*_*nf*_) and the solid (*θ*_*s*_) at *Ra* = 1000, *Ha* = 0, *ϕ* = 0.04.

**Figure 8:**
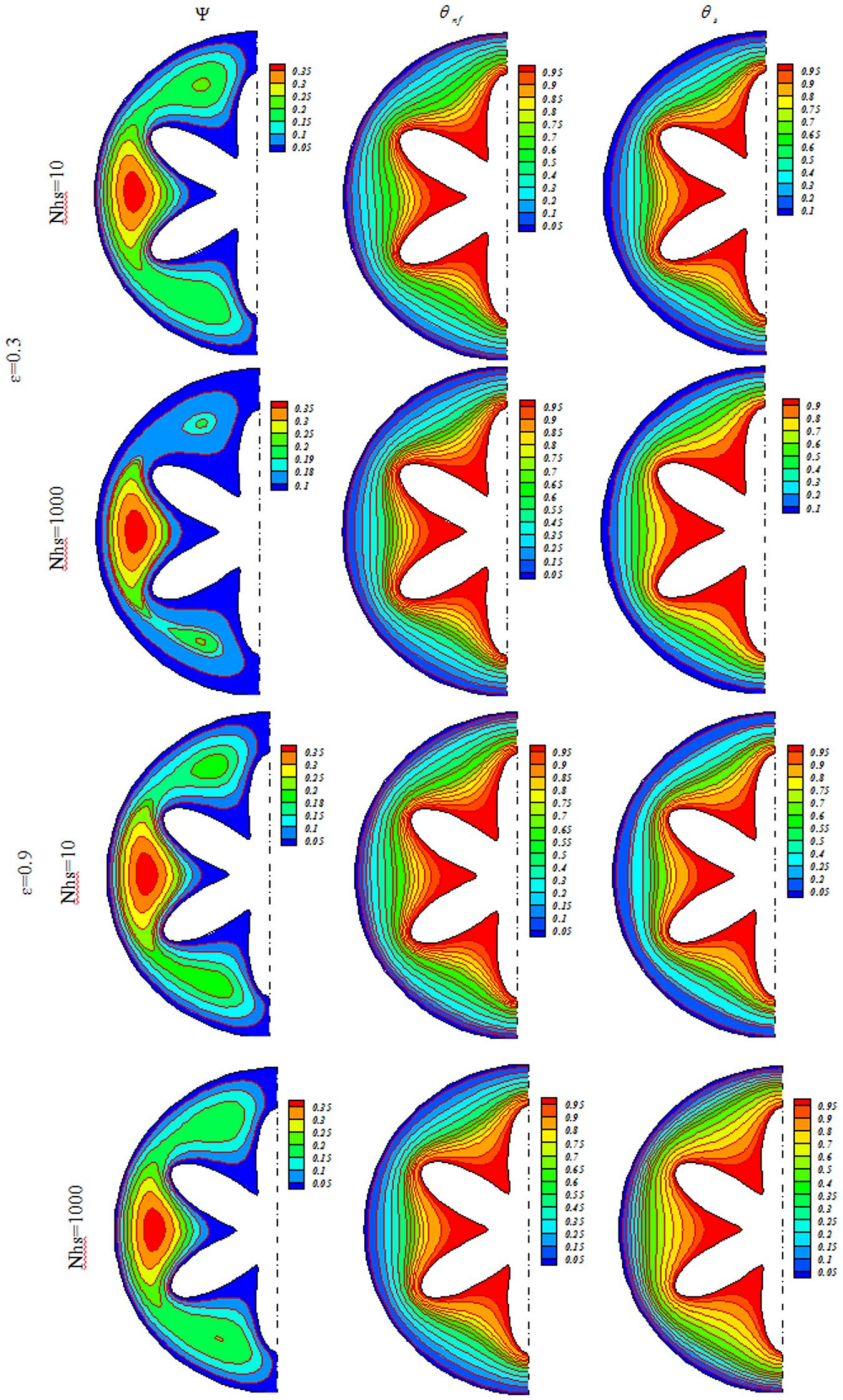
Streamlines (Ψ), isotherms for the nanofluid (*θ*_*nf*_) and the solid (*θ*_*s*_) at *Ra* = 1000, *Ha* = 20, *ϕ* = 0.04.

Influences of *Nhs*, *Ra*, *ε* and *Ha* on isotherms for solid (*θ*_*s*_), streamlines (Ψ) and nanofluid (*θ*_*nf*_) were depicted in Figs 3, 4, 5, 6, 7 and 8. When buoyancy force is weak, conduction mode is significant and the impacts of other variables are negligible. In this case, (*θ*_*nf*_) contours are similar to (*θ*_*s*_) contours. As Ra increase, thermal plume can be seen close to the vertical centerline and (*θ*_*nf*_) contours become more complex but (*θ*_*s*_) contours have no changes. Velocity of nanofluid decrease with augment of magnetic forces and the (*θ*_*nf*_) contours are stratified with enhance of *Ha*. (Ψ_max_) enhances with augment of *Nhs* due to stronger convective flow. As *ε* enhances, the pores volume through the enclosure augments. Therefore, convective flow becomes stronger. Besides, the impact of *ε* on isotherms is as same as *Nhs*.

**Figure 9:**
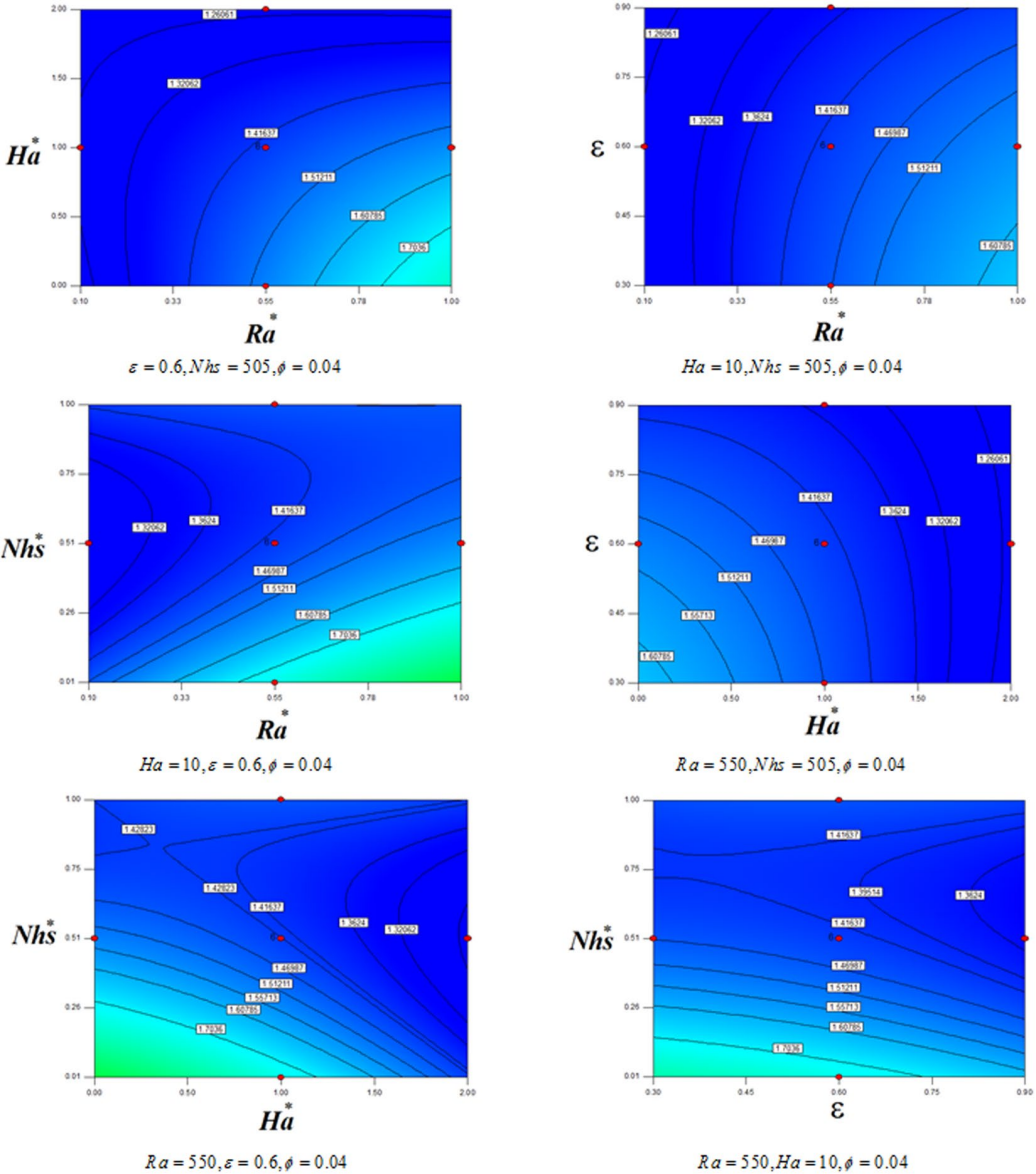
Contour plots for effects of *Ra*, *Ha*, *ε*, *Nhs* on average Nusselt number.

**Figure 10:**
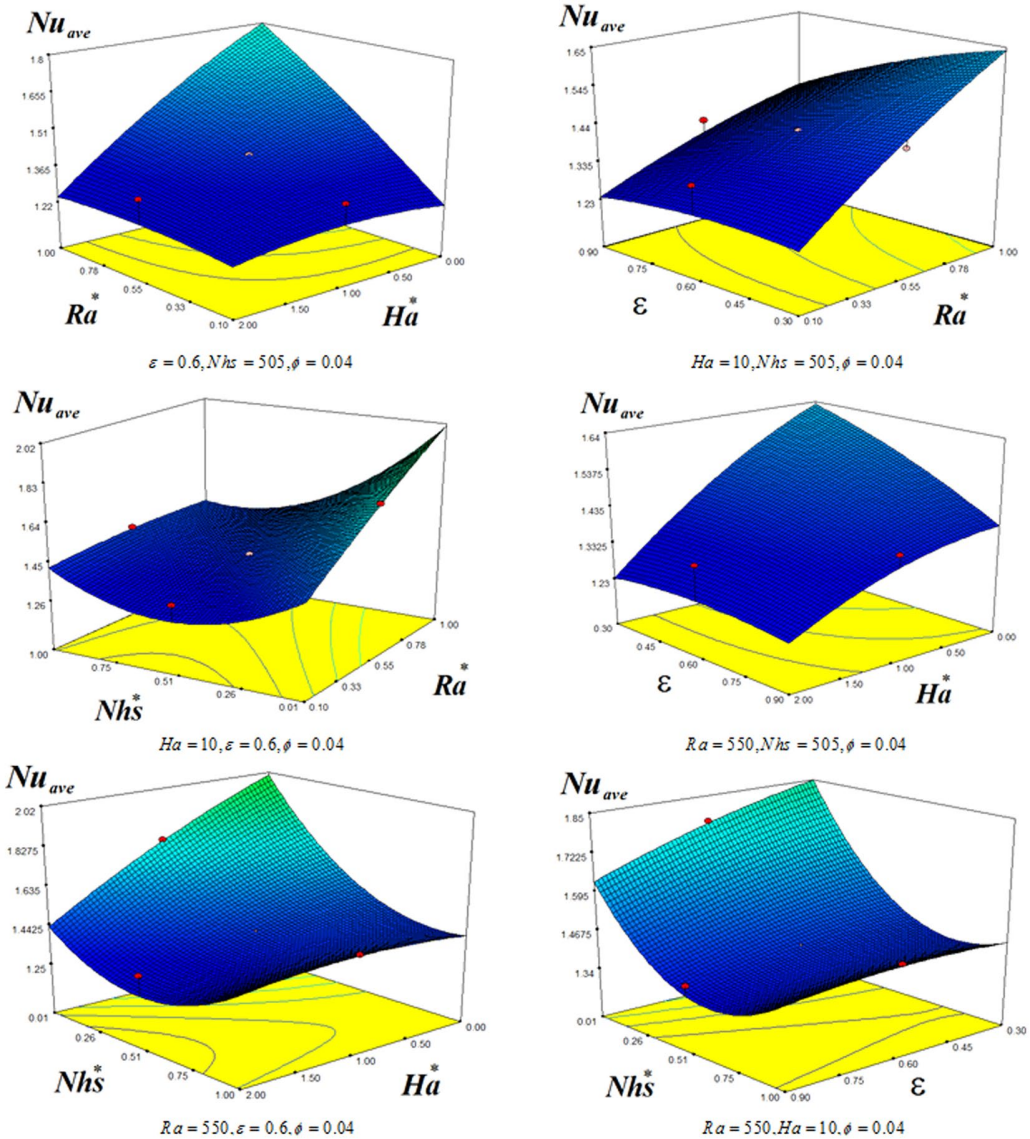
3D plots for effects of *Ra*, *Ha*, *ε*, *Nhs* on average Nusselt number.

Figures 9 and 10 depict the impact of *Nhs*, *ε*, *Ha* and *Ra*, on *Nu*_*ave*_. *Nu*_*ave*_ can calculate as:

21 $$\begin{array}{rcl}N{u}_{ave} & = & 1.64+1.3R{a}^{\ast }-0.16H{a}^{\ast }-0.12\varepsilon -1.24Nh{s}^{\ast }\\  &  & -0.3R{a}^{\ast }H{a}^{\ast }-0.34R{a}^{\ast }\,\varepsilon -0.55R{a}^{\ast }Nh{s}^{\ast }+0.19H{a}^{\ast }\varepsilon +0.28H{a}^{\ast }Nh{s}^{\ast }\\  &  & +\,0.34\varepsilon Nh{s}^{\ast }\\  &  & -0.08{(R{a}^{\ast })}^{2}+0.74{(Nh{s}^{\ast })}^{2}-0.036{(H{a}^{\ast })}^{2}-0.2{(\varepsilon )}^{2}\end{array}$$

Coefficient of determination is 0.98 for this correlation. Also, in this formula we have: Ha* = 0.1*Ha*, Nhs* = 0.001*Nhs*, Ra* = 0.001*Ra*. *Nu*_*ave*_ is a reducing function of *Nhs* because temperature gradient decreases with augment of this factor. Decreasing porosity makes Nu to enhance which is similar of Ha impact. *Nu*_*ave*_ decreases with reduce of Rayleigh number.

## Conclusions

In current research, migration of CuO nanoparticles is simulated via Non-equilibrium. Innovative method is applied to show the impacts of porosity, buoyancy, magnetic forces and the Nhs. Results show that *Nu*_*ave*_ reduces with augment of *Ha*, *ε*, *Nhs*. Convective flow enhances with increase of *Nhs* and *ε* while it reduces with enhance of magnetic force. When *Ra* = 1000, *ε* = 0.3, increasing *Nhs* leads to 17.06 percent decrement of *Nu*_*ave*_ in absence of magnetic field. Impact of *Nhs* is negligible in existence of Lorenz force. Also *Nu*_*ave*_ decreases about 53% with augment of Hartmann number.

## Electronic supplementary material

SUPPLEMENTARY INFO
