# Supplementary material for: Non-equilibrium Model for Nanofluid Free Convection Inside a Porous Cavity Considering Lorentz Forces
Source: Sci Rep. 2018 Nov 15;8:16881. doi: 10.1038/s41598-018-33079-6 (PMC6237777; doi:10.1038/s41598-018-33079-6)
Supplement: Supplementary file 1 — SUPPLEMENTARY INFO [file 41598_2018_33079_MOESM1_ESM.doc]

**Non-equilibrium model for nanofluid free convection inside a porous cavity considering Lorentz forces**

M. Sheikholeslami^b^, Ilyas Khan^a^,*

*^a^Faculty of Mathematics and Statistics, Ton Duc Thang University, Ho Chi Minh City, Vietnam.*

*^b^Department of Mechanical Engineering, Babol Noshirvani University of Technology, Babol, IRAN*

*Corresponding Author:

Emails: ilyaskhan@tdt.edu.vn (Ilyas Khan),

First author:

mohsen.sheikholeslami@nit.ac.ir (M. Sheikholeslami)

Collected figures

Fig. 1. (a) Geometry and the boundary conditions with (b) A sample triangular element and its corresponding control volume.

Fig. 2. Comparison of the present solution with previous work (Kim et al. [24]) for different Rayleigh numbers when Ra= 10^5^, Pr=0.7; (b) Comparison of the temperature on axial midline between the present results and numerical results obtained by Khanafer et al. [25] for$Gr=10^{4}$, and$\Pr=6.2\left(Cu-Water\right)$.

Fig. 3. Streamlines $\left(\Psi\right)$, isotherms for the nanofluid $\left(\theta_{nf}\right)$and the solid $\left(\theta_{s}\right)$at

Fig. 4. Streamlines $\left(\Psi\right)$, isotherms for the nanofluid $\left(\theta_{nf}\right)$and the solid $\left(\theta_{s}\right)$at

Fig. 5. Streamlines $\left(\Psi\right)$, isotherms for the nanofluid $\left(\theta_{nf}\right)$and the solid $\left(\theta_{s}\right)$at

Fig. 6. Streamlines $\left(\Psi\right)$, isotherms for the nanofluid $\left(\theta_{nf}\right)$and the solid $\left(\theta_{s}\right)$at

Fig. 7. Streamlines $\left(\Psi\right)$, isotherms for the nanofluid $\left(\theta_{nf}\right)$and the solid $\left(\theta_{s}\right)$at

Fig. 8. Streamlines $\left(\Psi\right)$, isotherms for the nanofluid $\left(\theta_{nf}\right)$and the solid $\left(\theta_{s}\right)$at

Fig. 9. Effects of $Ra,Ha,\varepsilon,Nhs$ on average Nusselt number.
